# Characterization of Zur-dependent genes and direct Zur targets in *Yersinia pestis*

**DOI:** 10.1186/1471-2180-9-128

**Published:** 2009-06-25

**Authors:** Yingli Li, Yefeng Qiu, He Gao, Zhaobiao Guo, Yanping Han, Yajun Song, Zongmin Du, Xiaoyi Wang, Dongsheng Zhou, Ruifu Yang

**Affiliations:** 1 State Key Laboratory of Pathogen and Biosecurity, Beijing Institute of Microbiology and Epidemiology, Beijing 100071, PR China; 2 Laboratory Animal Center, Academy of Military Medical Sciences, Beijing 100071, PR China

## Abstract

**Background:**

The zinc uptake regulator Zur is a Zn^2+^-sensing metalloregulatory protein involved in the maintenance of bacterial zinc homeostasis. Up to now, regulation of zinc homeostasis by Zur is poorly understood in *Y. pestis*.

**Results:**

We constructed a *zur *null mutant of *Y. pestis *biovar *microtus *strain 201. Microarray expression analysis disclosed a set of 154 Zur-dependent genes of *Y. pestis *upon exposure to zinc rich condition. Real-time reverse transcription (RT)-PCR was subsequently used to validate the microarray data. Based on the 154 Zur-dependent genes, predicted regulatory Zur motifs were used to screen for potential direct Zur targets including three putative operons *znuA, znuCB *and *ykgM*-*RpmJ2*. The LacZ reporter fusion analysis verified that Zur greatly repressed the promoter activity of the above three operons. The subsequent electrophoretic mobility shift assay (EMSA) demonstrated that a purified Zur protein was able to bind to the promoter regions of the above three operons. The DNase I footprinting was used to identify the Zur binding sites for the above three operons, verifying the Zur box sequence as predicted previously in γ-Proteobacteria. The primer extension assay was further used to determine the transcription start sites for the above three operons and to localize the -10 and -35 elements. Zur binding sites overlapped the -10 sequence of its target promoters, which was consistent with the previous observation that Zur binding would block the entry of the RNA polymerase to repress the transcription of its target genes.

**Conclusion:**

Zur as a repressor directly controls the transcription of *znuA, znuCB *and *ykgM*-*RpmJ2 *in *Y. pestis *by employing a conserved mechanism of Zur-promoter DNA association as observed in γ-Proteobacteria. Zur contributes to zinc homeostasis in *Y. pestis *likely through transcriptional repression of the high-affinity zinc uptake system ZnuACB and two alternative ribosomal proteins YkgM and RpmJ2.

## Background

Zinc is an essential trace element for a large number of enzymes and proteins in bacteria, but it can be toxic at high levels. It is therefore crucial that intracellular zinc level over a small concentration range must be tightly regulated [1-3]. Bacterial zinc homeostasis is achieved mainly by the coordinated expression of zinc uptake and export systems that are separately regulated by their own regulators [1-3].

Bacteria have evolved at least three types of Zn^2+ ^export systems [2,3] to protect cells from high toxic Zn^2+ ^concentrations, namely cation diffusion facilitators (e.g. CzcD in *Alcaligenes eutrophus*), RND type exporters (e.g. CzcABC in *A. eutrophus*), and P-type ATPases (e.g. ZntA in *Escherichia coli*). CzcD, CzcABC and ZntA are regulated by an ArsR-like repressor CzrA [4], a two-component system CzcR/S [5], and a MerR-family regulator ZntR [6], respectively.

Zinc ions are transported into the cytoplasm via high- and low-affinity zinc uptake systems, which are represented by ZnuABC of *E. coli *[7] and YciABC of *Bacillus subtilis *[8,9], respectively. A broad set of zinc uptake systems including ZnuABC and YciABC are regulated by the zinc uptake regulator Zur that is a homologous to the well-known Fur family of metal-dependent regulators [1].

*Yersinia pestis *is the causative agent of plague that is a zoonotic disease primarily affecting rodents [10]. Maintenance of plague in nature is primarily dependent upon cyclic transmission between fleas and rodents [10]. *Y. pestis *possesses its potential to attack humans, and the human infection usually occurs with the transmission of the pathogen from animals by the biting of an infected flea, but this deadly disease can be transmitted from person to person by respiratory route. *Y. pestis *can remain viable and fully virulent after 40 weeks in soil [11]. Thus, soil appears a potential telluric reservoir for *Y. pestis*, which could represent an alternative mechanism for maintenance of plague [11]. Zinc homeostasis should be crucial for survival of *Y. pestis *in fleas, rodents and soil.

Up to now, regulation of zinc homeostasis by Zur is poorly understood in *Y. pestis*. In this study, we constructed a *zur *null mutant of *Y. pestis *biovar *Microtus *strain 201, and compared its global gene expression profile to that of the parental strain by using cDNA microarray, identifying a total of 154 Zur-dependent genes. Three genes or operons, namely *znuA, znuCB *and *ykgM *were further identified as direct Zur targets. Subsequent determination of transcription start sites, predicted -10/-35 elements, and Zur binding sites enabled the mapping of Zur-DNA interactions for these three genes. This study confirmed that *Y. pestis *Zur employed a conserved regulatory mechanism observed in γ-Proteobacteria.

## Methods

### Bacterial strains

The wild-type (WT) *Y. pestis *biovar *Microtus *strain 201 is avirulent to humans but highly lethal to mice [12]. It was grown in Luria-Bertani (LB) broth or chemically defined TMH medium [13] at 26 or 37°C. *E. coli *strains BL21 (DE3) was grown in LB broth at 37°C. Antibiotics were added at the following concentrations when required: 100 μg/ml for ampicillin, and 50 μg/ml for kanamycin.

### Construction of the *zur *mutant

The *Y. pestis zur *mutant strain (*Δzur*) was generated by using the one-step inactivation method based on the lambda phage recombination system, as previously described by Datsenko and Wanner [14]. Briefly, the helper plasmid pKD46 was first transformed into *Y. pestis *201. The *zur*::*kana *mutagenic cassette was PCR amplified from plasmid pRS551 [15] with the primers *zur*-*k*-F and *zur*-*k*-R and transformed into strain 201/pKD46 (all the primers used in this study were listed in Additional file 1). Mutants were selected by plating electroporated cells on agar plates containing kanamycin. Colonies of resistant transformants were subsequently selected. Chromosomal integration of the mutagenic cassette was confirmed by PCR and sequencing using oligonucleotides external to the integrated cassette (data not shown). The mutants were incubated overnight at 37°C and then tested for the loss of the temperature-sensitive plasmid pKD46 by looking for ampicillin sensitivity. The elimination of the helper plasmid was verified by PCR (data not shown).

### Bacterial growth and RNA isolation

A chemically defined TMH medium [13] was used to cultivate strain 201. Both WT and *Δzur *were pre-cultivated at 26°C to the middle exponential growth phase (OD620 about 1.0) in TMH medium. The cell cultures were then diluted 1:20 in fresh TMH medium and grown at 26°C until an OD620 of about 1.0. Finally, 5 mM ZnCl_2 _was added into each cell culture to ensure zinc rich conditions. Growth was continued for 30 min at 26°C before harvested for total RNA isolation. This kind of treatment with Zn had no toxic effect on both WT and *Δzur*, according to the colony counting assay (Additional file 1).

Immediately before being harvested, bacterial cultures were mixed with two fold of RNAprotect Bacteria Reagent (Qiagen) to minimize RNA degradation. Total cellular RNA was isolated using the MasterPure™ RNA Purification kits (Epicenter). RNA quality was monitored by agarose gel electrophoresis and RNA quantity was measured by spectrophotometer.

### DNA microarray analysis

Gene expression profiles were compared between WT and *Δzur *by using a *Y. pestis *whole-genome cDNA microarray as described previously [12]. Briefly, RNA samples were isolated from four individual bacterial cultures, as biological replicates, for each strain. Total cellular RNA was isolated and then used to synthesize cDNA in the presence of aminoallyl-dUTP, genome directed primers (GDPs) and random hexamer primers [16]. The aminoallyl modified cDNA was then labelled with Cy5 or Cy3 dye. Microarray slides spotted in duplicate with 4005 PCR amplicons, representing about 95% of the non-redundant annotated genes of *Y. pestis *CO92 [17] and 91001 [18], were used for probe hybridization. The dual-fluorescently (Cy3 or Cy5 dye) labeled cDNA probes, for which the incorporated dye was reversed, were synthesized from the RNA samples of the four biological replicates, and then hybridized to four separated microarray slides, respectively. The scanning images were processed and the data was further analyzed by using GenePix Pro 4.1 software (Axon Instruments) combined with Microsoft Excel software. The normalized log_2 _ratio of the *Δzur*/WT signal for each spot was recorded. The averaged log_2 _ratio for each gene was finally calculated. Significant changes of gene expression were identified through the Significance Analysis of Microarrays (SAM) software (a Delta value of 1.397 and an estimated False Discovery Rate of 0%) [19].

### Computational analysis of Zur binding sites

The 500 bp promoter regions upstream the start codon of each Zur-dependent genes as revealed by cDNA microarray was retrieved with the '*retrieve-seq*' program [20]. A position count matrix was built from the predicted Zur binding sites in γ-Proteobacteria by using the *matrices-consensus *tool [20], and displayed by the *WebLogo *program to generate a sequence logo [21]. Following this, the *matrices-paster *tool [20] was used to match the Zur position count matrix within the above promoter regions.

### Real-time RT-PCR

Gene-specific primers were designed to produce a 150 to 200 bp amplicon for each gene (see Additional file 2 for primer sequences). The contaminated DNA in RNA samples was further removed by using the Amibion's DNA-free™ Kit. cDNAs were generated by using 5 μg of RNA and 3 μg of random hexamer primers. Using three independent cultures and RNA preparations, real-time RT-PCR was performed in triplicate as described previously through the LightCycler system (Roche) together with the SYBR Green master mix [22,23]. On the basis of the standard curves of 16S rRNA expression, the relative mRNA level was determined by calculating the threshold cycle (ΔCt) of each gene by the classic ΔCt method. Negative controls were performed by using 'cDNA' generated without reverse transcriptase as templates. Reactions containing primer pairs without template were also included as blank controls. The 16S rRNA gene was used as an internal control to normalize all the other genes. The transcriptional variation between WT and *Δzur *was calculated for each gene. A mean ratio of two was taken as the cutoff of statistical significance.

### Overproduction and purification of *Y. pestis *Zur protein

The 537 bp entire coding region of *zur *gene was amplified by primer Zur-P-F and Zur-P-R from *Y. pestis *201 (see Additional file 2 for primer sequences) and cloned directionally into the *BamHI *and *HindIII *sites of plasmid pET24a (Novagen), which was verified by DNA sequencing. The stop codon was introduced in the reverse primer to make sure that the expressed Zur did not contain His-tag. The resulted recombinant plasmid was transformed into *E. coli *BL21 (DE3). For overproduction of Zur, an overnight culture from a single colony was used to inoculate 200 milliliter of LB medium. Cells were grown with vigorous shaking at 37°C to an optical density at 620 nm (OD620) of 0.8 and were induced with 1 mM IPTG (isopropyl-β-D-thiogalactoside) for 6 h at 37°C. For purification, harvested cells were treated with BugBuster^® ^Protein Extraction Reagent (Novagen). Inclusion bodies were recovered by centrifugation and washed twice with the same reagent. The Zur protein was renaturated and then concentrated to a final concentration of about 0.6 mg/ml with the Amicon Ultra-15 (Millipore). The protein purity was verified by SDS-PAGE with silver staining. All steps after cell harvest were performed at 4°C, and the purified Zur protein was stored at -80°C.

### Gel mobility shift assay (EMSA)

Primers were designed to amplify the DNA region upstream of the start codon of each gene tested (see Additional file 2 for primer sequences). EMSA was performed by using the Gel Shift Assay Systems (Promega) [22,23]. The 5' ends of DNA were labeled using [γ-32P] ATP and T4 polynucleotide kinase. DNA binding was performed in a 10 μl reaction volume containing binding buffer [20 mM Tris-HCl (pH 8.0), 50 mM KCl, 1 mM DTT, 5% glycerol, 0.05 mg/ml poly-(dI-dC) and 100 μM ZnCl_2_], labeled DNA and various concentrations of the Zur protein. We still included three controls in each EMSA experiment: i) specific DNA competitor (unlabeled promoter region of the same gene); ii) nonspecific DNA competitor [unlabeled promoter region of the specific gene without the predicted binding site. one of the negative controls]; and iii) nonspecific protein competitor (rabbit anti-F1-protein polyclonal antibody). After incubation at room temperature for 30 min, the products were loaded onto a native 4% (w/v) polyacrylamide gel and electrophoresed in 0.5×TBE buffer for about 30 min at 220 V. Radioactive species were detected by autoradiography after exposure to Kodak film at -70°C.

### DNase I footprinting

The promoter DNA region was prepared by PCR amplification performed with the promoter-specific primer pairs (see Additional file 2 for primer sequences), including a 5'-^32^P-labeled primer (either forward or reverse) and its nonlabelled counterpart. The PCR products were purified by using MinElute reaction cleanup columns (Qiagen). Increasing amount of Zur was incubated with the labeled DNA fragment (2 to 5 pmol) for 30 min at room temperature in a final volume of 10 μl containing binding buffer same as EMSA [22,23]. Before DNA digestion, 10 μl of Ca^2+^/Mg^2+ ^solution (5 mM CaCl_2 _and 10 mM MgCl_2_) was added, followed by incubation for 1 min at room temperature. Then, the optimized RQ1 RNase-Free DNase I (Promega) was added to the reaction mixture, and the mixture was incubated at room temperature for 50 to 90 s. The cleavage reaction was stopped by adding 9 μl of the stop solution (200 mM NaCl, 30 mM EDTA and 1% SDS) followed by DNA extraction and precipitation. The partially digested DNA samples were then analyzed in a 6% polyacrylamide/8M urea gel. Protected regions were identified by comparison with the sequence ladders. For sequencing, the fmol^® ^DNA Cycle Sequencing System (Promega) was used. The result was detected by autoradiography (Kodak film).

### Primer extension assay

For the primer extension assay [22,23], about 10 μg of total RNA from each strain was annealed with 1 pmol of [γ-^32^P] end-labeled reverse primer (see Additional file 2 for primer sequences). The extended reverse transcripts were generated as described in the protocol for Primer Extension System-AMV Reverse Transcriptase (Promega). The yield of each primer extension product would indicate the mRNA expression level of the corresponding gene in each strain, and further could be employed to map the 5' terminus of RNA transcript for each gene. The same labeled primer was also used for sequencing with the fmol^® ^DNA Cycle Sequencing System (Promega). The primer extension products and sequencing materials were concentrated and analyzed by 8 M urea-6% polyacrylamide gel electrophoresis. The result was detected by autoradiography (Kodak film).

### *LacZ *reporter fusion and β-Galactosidase assay

The 500 to 600 bp promoter regions upstream the *znuA*, *znuCB*, and *ykgM *genes were obtained by PCR with the Takara ExTaq™ DNA Polymerase using *Y. pestis *201 genome DNA as the template (see Additional file 2 for primer sequences). PCR fragments were then cloned directionally into the *SmaI *(or *EcoRI*)and *BamHI *sites of plasmid pRS551 [15], which contains a promotorless *lacZ *reporter gene. Correct cloning was verified by DNA sequencing. Both WT and *Δzur *were transformed with the recombinant plasmids and grown as described in microarray analysis. The empty plasmid pRS551 was also introduced into both strains as negative control. β-Galactosidase activity was measured on cellular extracts by using the β-Galactosidase Enzyme Assay System (Promega) [22]. Assays were performed in triplicate.

## Results

### Identification of Zur-regulated genes by cDNA microarray

By the standard dual-fluorescent microarray hybridization experiments, mRNA level of each gene was compared between WT and *Δzur *upon exposure to zinc rich conditions. Totally, the transcription of 154 genes was found to be affected by the *zur *disruption. Among them, 90 genes were down-regulated in *Δzur*, while 64 genes up-regulated. According to the genome annotation of *Y. pestis *CO92, these Zur-dependent genes were distributed in 15 functional categories (Additional file 3). Their products included regulators, membrane-related proteins, transport/binding proteins, biosynthesis and metabolism related proteins and lots of unknown proteins. Additional file 4 showed the complete list of differentially regulated genes, giving an overall picture of the alteration of the global gene transcription pattern of *Y. pestis *affected by Zur with sufficient zinc. The microarray data (GSE15183) had been deposited in Gene Expression Omnibus (GEO).

### Validation of microarray data by Real-time RT-PCR

Microarray results are influenced by various factors, and thereby should be validated by at least one traditional method. Accordingly, the real-time quantitative RT-PCR, using RNA preparations as described in the microarray analysis, was performed to validate the microarray data. Based on gene classification, genomic location and transcriptional changes, 17 genes were chosen for RT-PCR (Additional file 5). The log-transformed change in relative quantity of mRNA level between WT and *Δzur *was calculated for each gene. The resulting real-time RT-PCR data were then plotted against the average log ratio values obtained by microarray analysis. There was a strong positive correlation (R^2 ^= 0.796) between the two techniques (Additional file 5). It should be noted that these 17 genes gave a 100% consistency for differential regulation between microarray and RT-PCR data, confirming the reliability of our microarray data.

### Characterization of DNA-binding ability of Zur by EMSA

**Figure 1:**
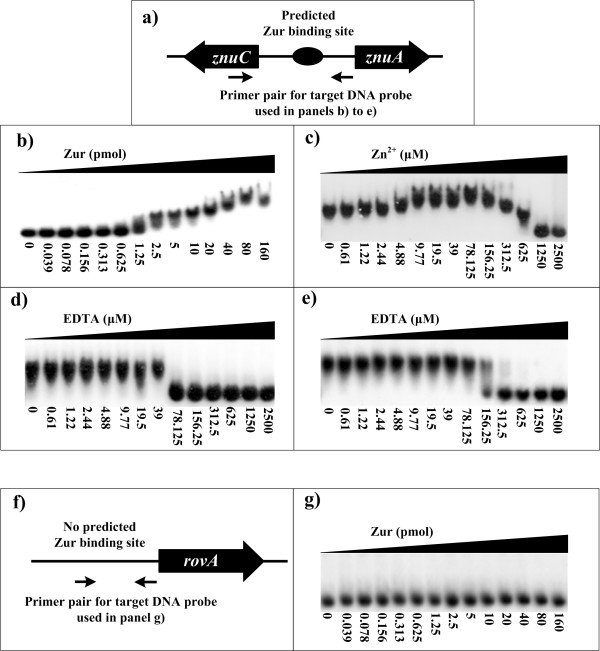
**DNA binding ability of Zur**. The upstream region of *znuA *(panel a) or *rovA *(f), with or without a predicted Zur binding site, respectively, was amplified by PCR and used as target DNA probe in EMSA. For EMSA, the [γ-^32^P]-labeled target DNA probes (1000 to 2000 c.p.m/μl) were incubated with the Zur protein in the presence or absence of 100 μM ZnCl_2_. Increasing amounts of Zur (b and g), ZnCl_2_(c), or EDTA (d and e) were employed. The mixtures were directly subjected to 4% polyacrylamide gel electrophoresis. The *rovA *gene was used as negative control.

We prepared a recombinant *Y. pestis *Zur protein by overproducing it in *E. coli *and examined its DNA-binding activity by EMSA (Fig. 1). Increasing amounts (from 0 to 160 pmol) of the purified Zur protein were incubated with 10 fmol of^32^P-labeled *znuA *promoter region (it contained a strongly predicted Zur binding site; see Fig. 1a) in the presence of 100 μM ZnCl_2 _(Fig. 1b). From 1.25 pmol of Zur, the Zur-DNA complex (i.e. gel retardation) emerged; with the Zur amount increased, gel retardation appeared more and more heavily and reached to the peak at 80 pmol of Zur.

It should be noted that the target DNA was progressively and continuously retarded (i.e. a gradual increase was observed in the gel mobility shift) with the increase in Zur concentration, and that 80 pmol Zur appeared to be a maximum for DNA binding (Fig. 1b). These results could be due to a second lower affinity binding site recognized by Zur at higher concentrations. Alternatively, like another regulator Fur [22], larger amounts of Zur proteins in the buffered environments would promote the formation of much more dimmers or even polymers, and thus there might be multiple Zur molecules bound to a single DNA site.

In assaying EMSA reactions containing either no zinc or increasing concentrations of zinc (from 0.61 to 2500 μM), 5 pmol of Zur was incubated with 10 fmol labeled *znuA *promoter region (Fig. 1c). With zinc concentrations increased, gel retardation occurred more and more heavily and reach the peak at 78 μM; since then, the efficacy of gel retardation decreased gradually, and a complete inhibition of Zur-DNA binding was observed when zinc concentration arising to 1250 μM. Accordingly, an optimized concentration of zinc at 100 μM was proposed for EMSA. Zur bound to target DNA even without added zinc, which might be due to the contamination of trace amount of Zn or other bivalent metal ions in the EMSA reactions, or due to the fact that the purified Zur protein might already contain some bound zinc with it.

To further validate the effect of zinc, with 5 pmol of Zur and 10 fmol of target DNA, EDTA at increasing concentrations (from 0.61 to 2500 μM) was added into different EMSA reactions respectively, so as to chelate zinc or other contaminated bivalent metal ions in the reaction mixture (Fig. 1d and 1e). The complete inhibition of Zur-DNA binding occurred from 78 μM EDTA without addition of zinc (Fig. 1d), while that occurred from 312.5 μM EDTA when 100 μM zinc was added (Fig. 1e).

The above results indicated that either zinc or Zur within a certain range of amounts was crucial for the Zur-DNA recognition. Generally, contaminated zinc or other bivalent metal ions was enough to ensure the Zur-DNA recognition in EMSA, but it would be promoted by addition of appropriate amounts of zinc into the reaction mixture.

To confirm the specificity of Zur-DNA association in EMSA, the EMSA experiments still included a *rovA *upstream DNA fragment for which no predicted Zur binding site was found (Table 1 and Fig. 1f). The negative EMSA results were observed, even though the Zur protein was increased to 160 pmol in a single reaction mixture (Fig. 1g).

**Table 1:** Genes tested in computational and biochemical assays

Gene ID	Gene	Computational marching of the Zur consensus			Position of DNA fragment used	
			
		Position §	Sequence	Score	EMSA	Footprinting
YPO3134	*ykgM*	-34 to -16	GATGTTACATTATAACATA	15.6	-134 to +102	-134 to +102
YPO2060	*znuC*	-45 to -27	AGCGTAATATTATAACATT	12.5	-185 to +52	-142 to +52
YPO2061	*znuA*	-49 to -31	AATGTTATAATATTACGCT	12.5	-158 to +67	-142 to +52

YPO1963	*astA*	-44 to -26	AAAGTTACGTCGTAACGTT	8.2	-165 to +124	-165 to +124
YPO1962	*astC*	-478 to -460	AATATTATTACATAACCGT	4.4	-498 to -2	---
YPO2374	*rovA*	---	---	0	-493 to -87	---
YPO2367	*gst*	---	---	0	---	-571 to -217

§, The numbers indicated the nucleotide position upstream the start code of each gene tested.

In all of the following EMSA experiments, 10 fmol of target DNA and 100 μM zinc without addition of EDTA were used in the reaction mixture.

### Screening for potential direct Zur targets by computational promoter analysis

**Figure 2:**
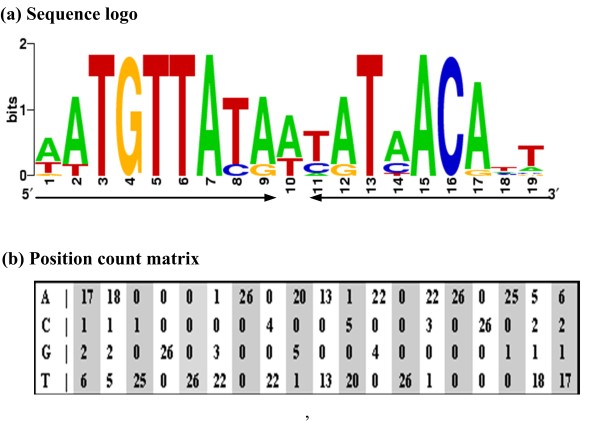
**The Zur regulatory consensus in γ-Proteobacteria**. (a) Original putative Zur binding sites were derived from Panina et al's study [29]. They were predicted from 13 genes in γ-Proteobacteria including *E. coli*, *Klebsiella pneumoniae*, *Salmonella typhi*, *Y. pestis*, and *Vibrio cholerae*, by the comparative genomics analysis [29]. Both coding and non-coding sequences of the above Zur sites were trimmed into 19 bp inverted repeat sequences and then aligned to generate a sequence-logo with a Zur box sequence (AATGTTATAWTATAACATT). (b) A position count matrix was generated as well from the alignment, where each row represented a position and each column a nucleotide. This matrix was subsequently used for the computational pattern matching analysis.

We further performed computational pattern matching analysis to predict direct Zur targets from the Zur-dependent genes disclosed by microarray. The regulatory consensus elements of Zur were analyzed (Fig. 2), and a position count matrix (Fig. 2c) was generated to statistically represent the conserved signals recognized by Zur, and subsequently used to screen for the potential Zur binding sites within the promoter sequences of the Zur-dependent genes uncovered by cDNA microarray. This analysis generated a score value for each promoter sequence, and the larger numbers of these scores would corresponded to the more highly consensus-like sequences in the promoters, i.e., the higher probability of Zur direct binding [20].

Four genes (*ykgM*, *znuC*, *znuA *and *astA*) giving the largest score values (Table 1) were picked out for further investigation. The former three genes represent the first genes of three distinct putative operons, namely *ykgM-rpmJ2, znuCB *and *znuA*, respectively. *ykgM *and *rpmJ2 *encoded ribosomal proteins, while *znuA*, *znuC *and *znuB *encoded the Zn^2+ ^uptake system ZnuABC. The *znuCB *and *znuA *operons were transcribed with opposite direction and separated by a short intergenic region (73 bp in length in *Y. pestis*) [17]. *astA *is the second gene of the *astCADBE *operon responsible for the arginine succinyltransferase pathway of arginine catabolism.

### Zur binds to DNA regions upstream *znuA*, *znuCB *and *ykgM-rpmJ2*

**Figure 3:**
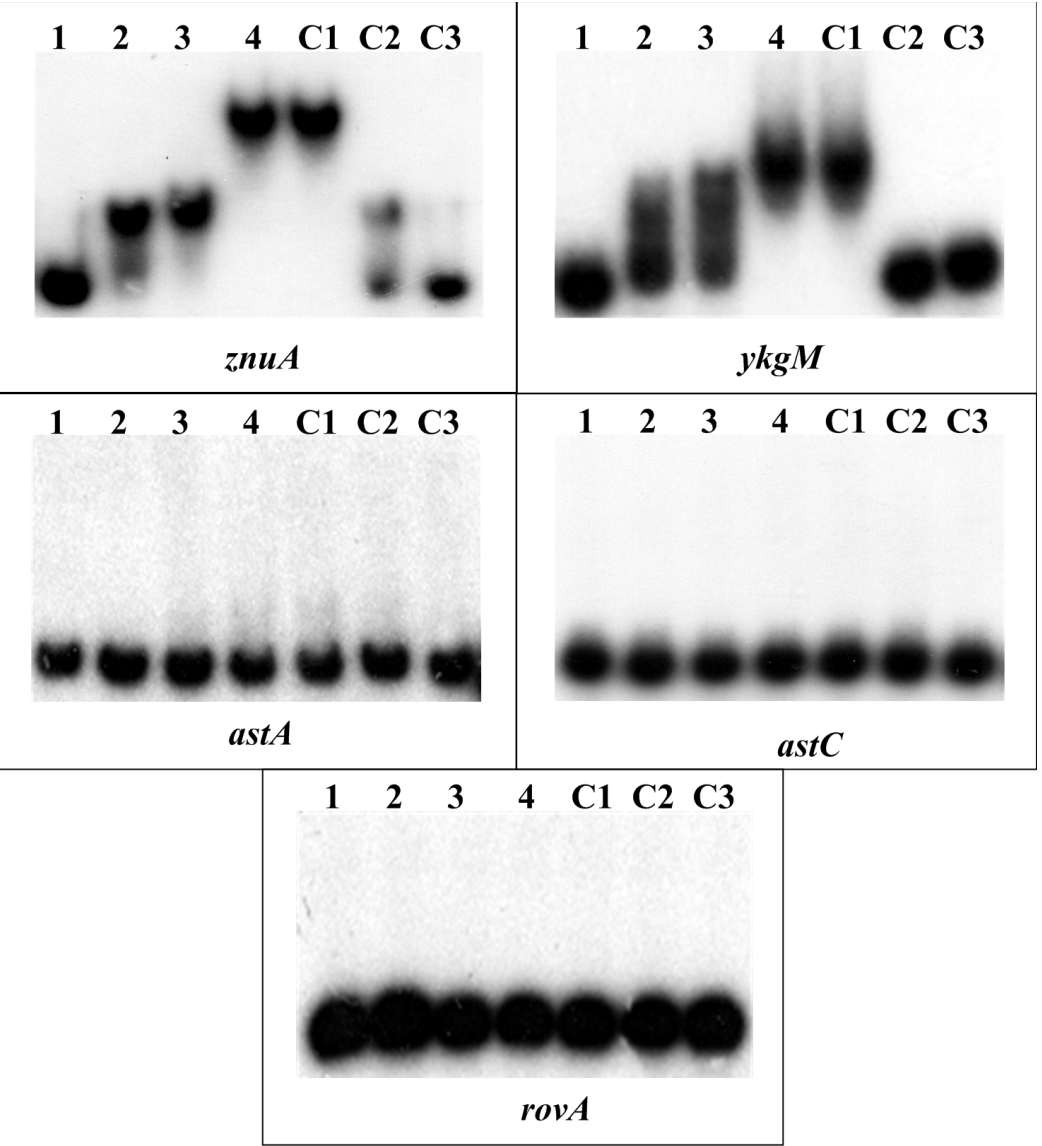
**Binding of Zur to the promoter regions of its potential target genes**. The [γ-^32^P]-labeled upstream region of each genes (10 fmol of target DNA probes) were incubated with the purified Zur protein in the presence of 100 μM ZnCl_2_. 0, 1.25, 2.5, 5, 5, 5 and 0 pmol of Zur were used in lanes 1 to 4 and C1 to C3, respectively. The mixtures were directly subjected to 4% polyacrylamide gel electrophoresis. For lanes 1 to 4, the retarded DNA band with decreased mobility turned up, which presumably represented the Zur-DNA complex. To confirm the specificity of the binding complexes, either a 200-fold molar excess of nonspecific competitor (2 pmol of unlabeled *znuA *DNA without its predicted binding region in lane C1) or a 200-fold molar excess of specific competitor (2 pmol of unlabeled target DNA probe in lane C2) was added to the binding mixture. 2 pmol of an unrelated protein, i.e., purified rabbit anti-F1 antibody, were included in lane C3. Both *znuA *and *znuC *gave positive EMSA results. Since these two genes had overlapped upstream regions and shared a single predicted Zur site, the EMSA data of only *znuA *rather than *znuC *was presented herein.

The real-time RT-PCR validated that Zur repressed the first gene of each of the three operons, *znuA*, *znuCB *and *ykgM-rpmJ2 *(Additional file 5). Herein, the DNA regions upstream these first genes (generated as indicated in Fig. 1a) were subjective to EMSA. It was demonstrated that the purified Zur protein bound to each of these potential target promoter regions in a Zur dose-dependent manner *in vitro *(Fig. 3). Thus, a direct association of Zur with the promoter regions of *znuA, znuCB *and *ykgM-rpmJ2 *was detected.

The EMSA experiments still included three additional genes, *astC*, *astA *and *rovA *(Fig. 3). As expected, the negative control *rovA *gave negative EMSA result. *astC *and *astA *were the first and second genes of the *astCADBE *operon, respectively. The whole operon was induced by Zur as determined by cDNA microarray, and real-time RT-PCR confirmed the up-regulation of *astC *by Zur (Additional file 5). *astA *gave a high score value (8.2) in the computational promoter analysis, while *astC *presented a very low value of 4.4 (Table 1). Both of *astC *and *astA *gave the negative EMSA results (Fig. 3). Herein, neither *astCADB *nor *astADB *was thought to be under the direct control of Zur by directly binding to a *cis*-acting element within corresponding upstream promoter region.

### Zur represses promoter activity of *znuA*, *znuCB *and *ykgM-rpmJ2*

To further validate the effect of Zur on the promoter activity of *znuCB*, *znuA *and *ykgM-rpmJ2*, we constructed the *znuC*::*lacZ*, *znuA*::*lacZ *and *ykgM*::*lacZ *fusion promoters each consisting of an upstream DNA of the corresponding gene, and then each of them was transformed into WT and *Δzur*, respectively. The β-galactosidase production of these *lacZ *fusions was measured in both WT and *Δzur*, which represented the promoter activity of the corresponding gene in each strain.

It should be noted that the *zur *mutation had an effect on the copy number of recombinant or empty pRS551 plasmid, and accordingly a normalized Miller unit was used to calculate the fold change in the activity of each fusion promoter in *Δzur *in relative to WT (Table 2). For each of the three genes, there was a significant increase of β-galactosidase activity in *Δzur *compared to WT when they grew in TMH with the addition of zinc. Thus, Zur repressed the promoter activities of *znuC*, *znuA *and *ykgM*. Taken all the above results together, it could be rational to say that *znuA*, *znuCB *and *ykgM-rpmJ2 *were under the direct and negative control of Zur.

**Table 2:** Promoter activity determined by LacZ reporter fusion analysis

LacZ fusion	Plasmid copy number (WT/*Δzur*)	Normalized Miller Units	Fold change (*Δzur*/WT)
WT-*znuC*	5.45 ± 0.73	6343.95 ± 237.68	2.60
*Δzur*-*znuC*		16507.10 ± 344.19	

WT-*znuA*	11.52 ± 0.92	12281.64 ± 428.30	7.77
*Δzur*-*znuA*		95498.09 ± 1962.30	

WT-*ykgM*	3.09 ± 0.88	118.64 ± 6.77	4.71
*Δzur*-*ykgM*		559.29 ± 28.14	

Notes: The promoter DNA regions upstream *znuC*, *znuA *and *ykgM *were cloned into the pRS551 plasmid, respectively, to fuse with the promoterless *lacZ *gene. β-Galactosidase activity (miller units) was detected to represent the promoter activity. Copy number of recombinant pRS551 was determined by real-time quantitative PCR, with the primers specific for the borne *lacA *gene. The detecting fold change of plasmid copy number was set to be 1 to generate a normalization factor that was subsequently used for generating the normalized fold change of promoter activity (miller units) in WT in relative to *Δzur*.

### Structural organization of Zur-dependent *znuCB, znuA *and *ykgM-rpmJ2 *promoters

**Figure 4:**
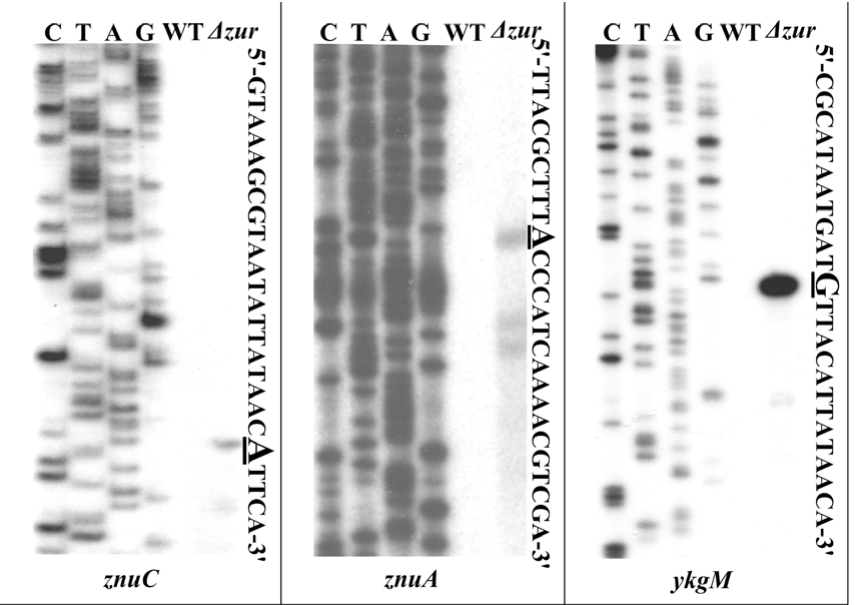
**Primer extension assays**. Primer extension assays were performed for *znuC*, *znuA *and *ykgM*, by using RNA isolated from the exponential-phase of both WT and *Δzur *grown in TMH medium with 5 mM of Zn^2+^. An oligonucleotide primer complementary to the RNA transcript of each gene was designed from a suitable position. The primer extension products were analyzed with 6% acrylamide sequencing gel. Lanes C, T, A and G represented the Sanger sequencing reactions. On the right side, DNA sequences were shown from the bottom (5') to the top (3'), and the transcription start sites were underlined.

Primer extension assay was performed to determine the transcription start sites of *znuC*, *znuA *and *ykgM *(Fig. 4). A strong primer extension product was detected for both *znuC *and *ykgM*, while three primer extension products were detected for *znuA*. Since the shorter extension products might represent the premature stops due to difficulties of polymerase in passing difficult sequences, only the longest product was chosen for the transcription start site of *znuA*. Accordingly, a transcription start site was identified for each of the three genes, and thereby a Zur-dependent promoter was transcribed for each of them. The nucleotide number of each transcription start site was taken as '+1', and the -10 and -35 core promoter elements recognized by sigma factor 70 were predicted upstream the transcription start sites.

**Figure 5:**
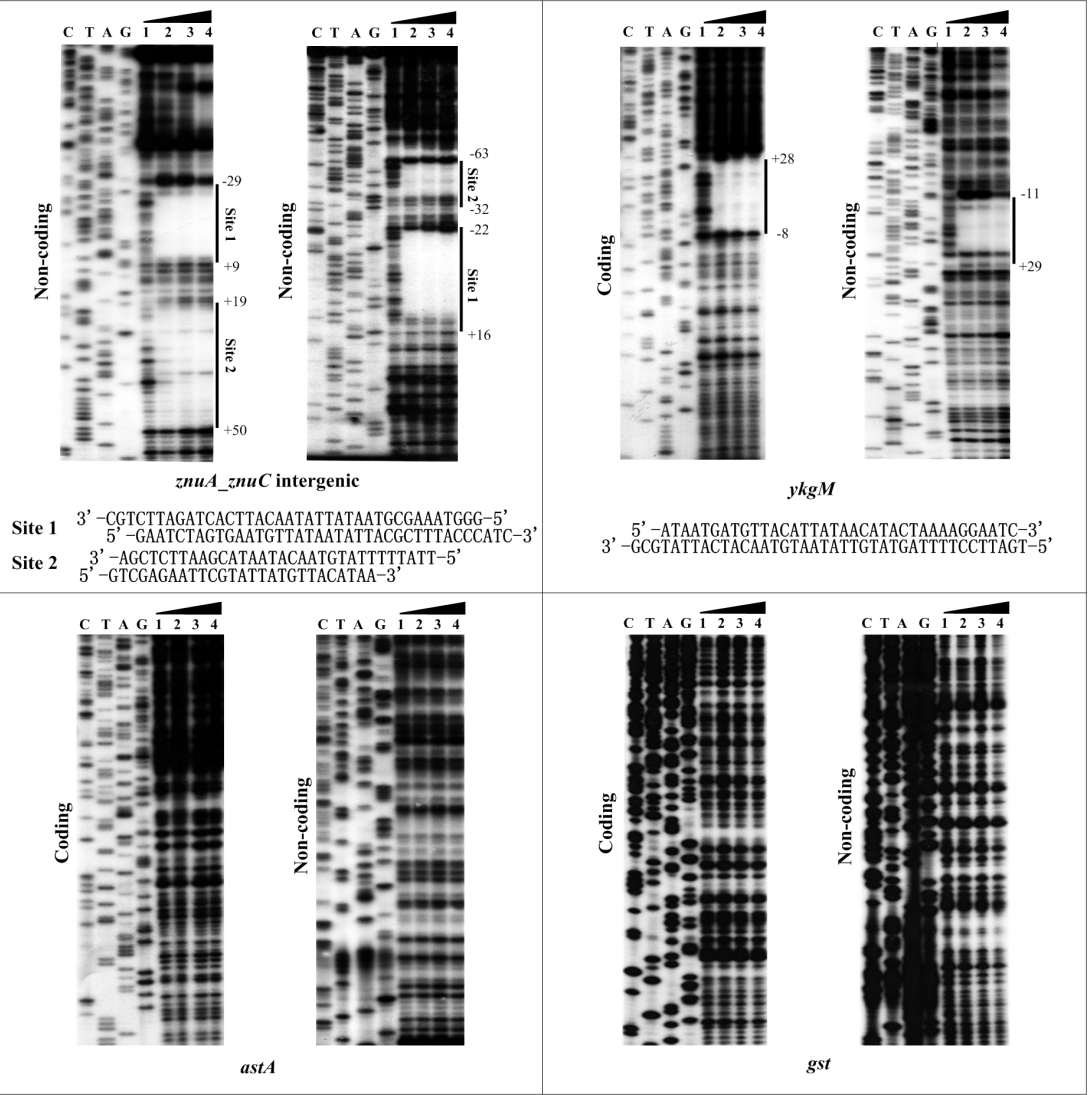
**DNase I footprinting assays**. Both the coding and noncoding strands of the promoter DNA fragments were generated by PCR. Labeled DNA probe was incubated with various amounts of purified Zur (lanes 1, 2, 3 and 4 contained 0, 2.5, 5 and 10 pmol, respectively). After partial digestion with DNase I, the resulting fragments were analyzed with 6% acrylamide sequencing gel. Lanes C, T, A and G represented the Sanger sequencing reactions. On the right side, the Zur protected regions were labeled with bold lines, and the footprint sequences were shown below. Positive and minus numbers flanking the bold lines indicate the nucleotide positions downstream and upstream the transcriptional site (taken as +1), respectively.

To precisely determine the Zur binding sites of *znuCB*, *znuA *and *ykgM-rpmJ2*, DNase I footprinting assay was performed in the presence of zinc (both coding and noncoding strands) (Fig. 5). DNase I footprinting results confirmed the binding of Zur to these promoter regions *in vitro*. Zur protected a distinct DNA region (i.e. Zur binding site) against DNase I digestion in a dose-dependent pattern for *ykgM *(Fig. 5). As expected, the Zur box was found in this footprint region. *znuCB *and *znuA *are transcribed with opposite direction. Two separated footprint regions (sites 1 and 2) were detected within the *znuCB*-*znuA *intergenic region. The Zur box was found in site 1 rather than site 2.

The DNase I footprinting assay still included two additional genes *astA *and *gst*. The *gst *upstream DNA region gave no predicted Zur site (Table 1), while EMSA indicated that Zur could not bind the *astA *promoter region *in vitro *(Fig. 3). As expected, no Zur-protected region was detected within the promoter DNA regions for both *astA *and *gst *(Fig. 5).

**Figure 6:**
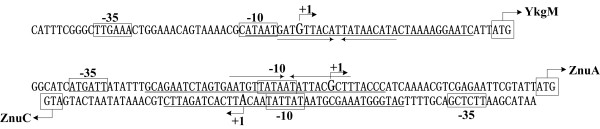
**Organization of Zur-dependent promoters for *znuC*, *znuA *and *ykgM***. The DNA sequences derived from the genomic data of *Y. pestis *CO92 and the start codon (ATG) of each gene was shown at the 3' terminus. The bent arrows indicated the transcription start sites and the corresponding nucleotide numbers were shown by taking the transcription start site as "+1". The predicted promoter -10 and/or -35 elements were boxed. Zur binding sites were underlined. The invert repeats in the Zur box was showed with two invert arrows.

The determination of Zur binding sites, transcription start sites, and core promoter elements (-10 and -35 regions) promoted us to depict the structural organization of Zur-activated *znuCB*, *znuA *and *ykgM-rpmJ2 *promoters (Fig. 6), giving a map of Zur-promoter DNA interaction for these genes.

## Discussion

### Global characterization of Zur-dependent genes

Zur senses the intracellular levels of zinc ions, and mediates a transcriptional response aimed at restoring homeostasis [1,7]. Under zinc-rich conditions, Zur binds the divalent zinc ion and inhibits the transcription of target genes. Under zinc-restricted conditions, Zur does not bind to the corresponding genes and the zinc homeostasis functions are expressed.

The microarray expression analysis is able to compare the expression profiles between a WT strain (Reference sample) and the isogenic mutant (Test sample) of Zur. Accordingly, the detecting Zur-dependent genes included various functional categories of genes, as characterized in a variety of bacteria including *B. subtilis *[9], *Mycobacterium tuberculosis *[24], *Streptococcus suis *[22] and *Xanthomonas campestris *[25]. In the present work, a total of 154 genes were found to be regulated by Zur in *Y. pestis*.

When a score value of 8 was taken as the cutoff, the computational pattern matching analysis revealed that only four Zur-dependent genes/operons (*ykgM-rpmJ2, znuCB, znuA *and *astA*) contained the predicted Zur binding sites within their upstream regions, and further EMSA experiments confirmed that Zur bound to the target promoters for the former three, rather than *astA *with a score value of 8.2 that was the lowest one compared to those of the other three. Thus, most of these differentially regulated genes were affected by Zur indirectly due to the following reasons [24]: i) the *zur *mutant could accumulate more zinc than the wild type, which could cause the transcriptional changes in some genes as a side-effect, and ii) Zur affected some regulatory genes and thus indirectly regulate downstream genes through these local regulators.

Remarkably, the most strongly Zur-repressed genes (Additional file 2) included *znuA, ykgM-rpmJ2*, *rovA *(a virulence-required regulator to induce *psaEF*),*psaEF *(a regulator to induce *psaABC*), *psaA *(the virulence determinant pH6 antigen), *ail *(YPO2190, a putative attachment invasion locus protein), YPO1343–1348 (transport/binding proteins) and YPO4018–4021 (phosphoribosyl transferase proteins). In addition to major zinc homeostasis functions (the zinc transport system ZnuABC, and two ribosomal proteins YkgM and RpmJ2; see below), several virulence-related genes (*rovA*, *psaEF*, *psaA *and *ail*) were greatly repressed by Zur under zinc-rich conditions. It was thought that *Y. pestis *responded to zinc limitations, and thereby modulated the expression of not only zinc homeostasis-related functions but also some virulence functions required for infection. The *in vivo *regulatory cascade between Zur and these virulence-related genes needs to be elucidated in *Y. pestis*.

### *Cis*-acting DNA consensus of the repressor Zur

Native Zur is a dimer, even in the absence of zinc or other metal ions [1,7]. Zur contains two zinc binding motifs, and binds at least two Zn^2+ ^per dimer specifically [1,7]. Mainly acting as a negative regulator, Zur with Zn^2+ ^as a cofactor binds to an consensus sequence (called 'Zur box') overlapping either the -35 region or the entire -10/-35 region of its target promoters, to block the entry of the RNA polymerase and thereby to repress the transcription of its target genes [24-28].

Computational comparative genomics analysis [29] identified the Zur box sequences of GAAATGTTATANTATAACATTTC for γ-proteobacteria, GTAATGTAATAACATTAC for the *Agrobacterium *group of α-proteobacteria, GATATGTTATAACATATC for the *Rhodobacter *group of α-proteobacteria, and TAAATCGTAATNATTACGATTTA for the *Bacillus *group of Gram-positive bacteria. The above Zur binding motifs differs from each other in nucleotide sequence, but all of them are about 20 bp AT-rich sequences and consist of two imperfect inverted repeat.

In the present study, a 19 bp palindrome sequence (AATGTTATANTATAACATT) was identified to be the Zur box in *Y. pestis*, which confirmed those predicted in γ-Proteobacteria (see above). In our previous study [12,22], the iron-responsive Fur regulon was characterized in *Y. pestis*. Fur and Zur represent the two members of the Fur-family regulators in *Y. pestis*. The *Y. pestis *Fur box sequence is a 9-1-9 inverted repeat (5'-AATGATAATNATTATCATT-3') [12,22]. The conserved signals recognized by Fur and Zur show a high level of similarity in nucleotide sequence [30].

### Direct Zur targets

As collectively identified in *E. coli *[26], *B. subtilis *[27,28], *M. tuberculosis *[24], *S. coelicolor *[31,32] and *X. campestri *[25], direct targets of the repressor Zur include primarily zinc transport systems (e.g. ZnuABC) and other membrane-associated transporters, protein secretion apparatus, metallochaperones, and a set of ribosomal proteins. The repressor Zur generally binds to a Zur box-like *cis*-acting DNA element within its target promoter regions (see above). Zur still acts as a direct activator of a Zn^2+ ^efflux pump in *X. campestris*; in this case, Zur binds to a 59 bp GC-rich sequence with a 20 bp imperfect inverted repeat overlapping the -35 to -10 sequence of its target promoter[25].

In the present work, Zur as a repressor directly regulated *znuA*, *zunBC *and *ykgM-rpmJ2 *in *Y. pestis*. Zur binds to the Zur box-like sequences overlapping the -10 region within the target promoters (Fig. 6), and thus *Y. pestis *Zur employed a conserved mechanism of Zur-promoter DNA association as observed in γ-Proteobacteria (see above).

### Regulation of zinc homeostasis by Zur

The high-affinity zinc uptake system ZnuABC belongs to the ABC transporter family and is composed of the periplasmic binding protein ZnuA, the ATPase ZnuC, and the integral membrane protein ZnuB [7]. Only in the presence of zinc or other divalent metal cations, Zur binds to a single *cis*-acting DNA element within the bidirectional promoter region of *znuA *and *znuCB *[24-26]. In this work, two separated DNase I footprint regions (sites 1 and 2) were detected within the *znuCB*-*znuA *intergenic region. The Zur box was found in only site 1 other than site 2. It was postulated that a Zur molecule might recognize the conserved Zur box (site 1) and further cooperatively associate with another Zur molecule to help the later one to bind to a less conserved (or completely different) binding site (site 2). Further reporter fusion experiments and/or *in vitr*o transcription assays, using *znuCB*-*znuA *intergenic promoter regions with different mutations/deletions within sites 1 and 2, should be done to elucidate the roles of site 1 and site 2 in Zur-mediated regulation of *znuCB *and *znuA*.

More than 50 ribosomal proteins together with three rRNAs (16S, 23S, and 5S rRNA) constitute the prokaryotic ribosome that is a molecular machine for protein biosynthesis. Most prokaryotic ribosomal proteins conserved highly, and their genes are assigned as single-copy genes in the genomes of many bacteria and are indispensable for cell viability. Some ribosomal protein genes (e.g. L36, L33, L31 and S14) have their paralogous pairs in many bacterial genomes, and it remains unclear why many bacteria possess these duplications in their genomes [33].

Zinc controls transcription of L36, L33, L31 and S14 [33]. Each paralogous pairs can be classified into two types; one type contains a CxxC zinc binding motif (generally a pair of conserved cysteines; designated C+), whereas the other does not (C-) [33]. The C- forms have lost the Zn ribbons in contrast to their original ribosomal proteins [33]. It was predicted that an ancient duplication of the C+ forms took place before the divergence of major bacterial lineages. Subsequently, loss of the C+ form or loss of the CxxC motif after the duplication generated the C-form) [33,34]. The C+ form is stable in cell when it contains a zinc ion bound to its CxxC motif [34,35].

The paralogous pairs of L31 protein are RpmE (C+) and YtiA (C-) in *B. subtilis *[34,35]. Expression of *ytiA *is repressed by Zur using zinc as its cofactor [34]. Liberation of RpmE from ribosome is triggered by the expression of *ytiA*, which is induced by the de-repression of Zur under zinc-deficient conditions [35]. The paralogous pairs of L31 protein are RpmE (YPO0111) and YkgM (YPO3134) in *Y. pestis*, while those of L36 protein are RpmJ (YPO0230) and RpmJ2 (YPO3135) [17]. YkgM and RpmJ2 are the C- forms of corresponding ribosomal proteins. *ykgM *and *rpmJ2 *constitutes a putative *ykgM*-*rpmJ2 *operon in *Y. pestis *[17]. It was shown herein that the *ykgM*-*rpmJ2 *operon was repressed by Zur. As expected, Zur bound to a Zur box-like element within the *ykgM *promoter region.

Almost all the L36, L33, L31, and S14 protein genes are regulated by zinc in *S. coelicolor*, and their C- paralogs was negatively regulated by Zur [31,32]. Similar findings have been reported in *M. tuberculosis *[24].

Taken the above together, a regulatory cascade was proposed herein on the basis of the previous notions [31-35]. Zinc was a key factor in controlling changes in the composition of L36, L33, L31 and S14 proteins in ribosome. Under zinc rich conditions, original L36, L33, L31 and S14 proteins (C+) bound with zinc ions were stable and functional in ribosome, and expression of their C- counterparts was repressed by Zur using zinc as its cofactor. Under zinc starvation conditions, these C+ proteins would not contain a zinc ion and would thus no longer be stable in the cell, while the zinc starvation would cause a de-repression of expression of their C- counterparts and would be associated with the ribosome instead of corresponding C+ proteins. The above alternation between C+ and C- ribosomal proteins might be helpful to increase the concentration of zinc ions available for other zinc-requiring proteins in the cell. Therefore, the above proposed regulatory cascade would contribute to bacterial zinc homeostasis under zinc-deficient conditions.

## Conclusion

A *zur *null mutant of *Y. pestis microtus *strain 201 was constructed in the present work. Microarray expression analysis disclosed a set of 154 Zur-dependent genes of *Y. pestis *upon exposure to zinc rich condition, and the microarray data was validated by real-time RT-PCR. Further biochemical assays, including LacZ reporter fusion, EMSA, DNase I footprinting, and primer extension, revealed that Zur as a repressor directly controlled the transcription of *znuA, znuCB *and *ykgM*-*RpmJ2 *in *Y. pestis *by employing a conserved mechanism of Zur-promoter DNA association as observed in γ-Proteobacteria. It was thought that Zur contributed to zinc homeostasis in *Y. pestis *through transcriptional repression of the high-affinity zinc uptake system ZnuACB and two alternative ribosomal proteins YkgM and RpmJ2.

## Authors' contributions

DZ and RY conceived the study and designed the experiments. YL performed all the experiments as well as data mining. YQ and HG contributed to LacZ reporter analysis, primer extension assay, and DNA binding assays. HG and ZG were involved in protein expression and purification. DZ and YH participated in microarray analysis. DZ, YS, ZD and XW assisted in computational analysis and figure construction. The manuscript was written by YL and DZ, and revised by RY. All the authors read and approved the final manuscript.

## Supplementary Material

Additional file 1 **Colony counting of WT and *Δzur *upon exposure to 5 mM Zn**. We performed colony counting of WT and *Δzur *upon exposure to 5 mM Zn for 30 min. The treatment with Zn had no toxic effect on both WT and *Δzur*. Click here for file

Additional file 2 Oligonucleiotide primers used in this study. Click here for file

Additional file 3 **Zur-regulated genes grouped by functional classification according to *Y. pestis *CO92 genome annotation**. Gene expression in *Δzur *was compared with that in the WT strain under Zn^2+ ^rich (5 mM) condition. The Zur-regulated genes were divided into various functional categories. The numbers of up- and down-regulated genes were represented for each functional group. Click here for file

Additional file 4 A complete list of Zur-regulated genes. Click here for file

Additional file 5 **Comparison of transcription measurements by microarray and real-time PCR assays**. The relative transcriptional levels for 17 genes selected from Supplementary Table S1 were determined by real-time RT-PCR. The log2 values were plotted against the microarray data log2 values. The correlation coefficient (R^2^) for comparison of the two datasets is 0.796. Click here for file
