# Supplementary material for: Characterization of Zur-dependent genes and direct Zur targets in Yersinia pestis
Source: BMC Microbiol. 2009 Jun 25;9:128. doi: 10.1186/1471-2180-9-128 (PMC2706843; doi:10.1186/1471-2180-9-128)
Supplement: Additional file 1 — Colony counting of WT and Δzur upon exposure to 5 mM Zn. We performed colony counting of WT and Δzur upon exposure to 5 mM Zn for 30 min. The treatment with Zn had no toxic effect on both WT and Δzur. [file 1471-2180-9-128-S1.doc]

**Colony counting of WT and *Δzur* upon exposure to 5 mM Zn**

| **Treatment** | |  | **CFU values (n=6)** | | | | | | | **P**  **value** |
| --- | --- | --- | --- | --- | --- | --- | --- | --- | --- | --- |
| **Strain** | **Added with** |  | **1** | **2** | **3** | **4** | **5** | **6** | **Mean ± SD** |  |
| WT | H_2_O |  | 127 | 191 | 120 | 108 | 111 | 144 | 134±31 | 0.44 |
|  | 5mM ZnCl_2_ |  | 102 | 128 | 114 | 152 | 168 | 121 | 131±25 |  |
| *Δzur* | H_2_O |  | 92 | 93 | 66 | 123 | 100 | 82 | 93±19 | 0.21 |
|  | 5mM ZnCl_2_ |  | 115 | 91 | 148 | 89 | 69 | 113 | 104±27 |  |

**Notes:** Both WT and *Δzur* were pre-cultivated at 26 ºC to the middle exponential growth phase (an OD_620_ about 1.0) in TMH. The cell cultures were then diluted 1:20 in fresh TMH medium and grown at 26 °C until an OD620 of 1.0. For each strain, the cell culture was split into six portions (each 3ml in a test tube) that were subjective to following treatments for 30 min at 26 °C: i) for each of three of them (three biological replicates), ZnCl_2_ was added to a final concentration of 5 mM to ensure zinc rich conditions; and iii) for each of the remaining three, H_2_O was added as the blank control. Serial dilutions of 200µl of the treating cell culture were plated onto BHI agar to calculate the numbers of CFU after the incubation at 26°C for 48h. Data from two agar plates (two technical replicates) for each treatment (at the 10^-6^ dilution) were collected. Thus, there were six data points in total for each treatment, and the averaged CFU values were calculated accordingly. Student’s one-tailed T test was performed for each strain to determine whether there was a significant difference (a P vale less than 0.05) between Zn treatment and blank control.
