# Supplementary material for: Characterization of Zur-dependent genes and direct Zur targets in Yersinia pestis
Source: BMC Microbiol. 2009 Jun 25;9:128. doi: 10.1186/1471-2180-9-128 (PMC2706843; doi:10.1186/1471-2180-9-128)
Supplement: Additional file 2 — Oligonucleiotide primers used in this study. [file 1471-2180-9-128-S2.doc]

**Oligonucleiotide primers used in this study**

| **Gene ID** | **Gene name** | **Real-time quantitative RT-PCR (Sense/antisense) (5'-3')** | **EMSA**  **(Sense/antisense) (5'-3')** | **primer extension**  **(antisense) (5'-3')** | **DNase I footprinting**  **(Sense/antisense) (5'-3')** | **LacZ reporter analysis**  **(Sense/antisense) (5'-3')** |
| --- | --- | --- | --- | --- | --- | --- |
| CO92-YPO0499 |  | TCAGTGAGCAAGCCAATAAGC/ |  |  |  |  |
|  |  | GGTTCTTCCGATTGAGGGTAG |  |  |  |  |
| CO92-YPO0625 |  | GGCGAGTTGAAATGTCTGATG/ |  |  |  |  |
|  |  | TGGATGTGTTGTTGTAAAGGAC |  |  |  |  |
| CO92-YPO0712 | *fleR* | GCAAGAGCAAGAAGTTGAACG/ |  |  |  |  |
|  |  | GATAGGCACCACGGATAAGC |  |  |  |  |
| CO92-YPO1231 | *pla2* | CGCACTGGCATTACTTATTCTG/ |  |  |  |  |
|  |  | ATGGGTGTATTGTTGGCTTCC |  |  |  |  |
| CO92-YPO1301 | *psaE* | GTCGTCTCCCCAACATCAC/ |  |  |  |  |
|  |  | TTCGGTGCTGCCATCATC |  |  |  |  |
| CO92-YPO1343 |  | GATGTTGATGGTCGTGAGGTC/ |  |  |  |  |
|  |  | ATTGGCTGGTCCGTAGGC |  |  |  |  |
| CO92-YPO1906 | *fyuA* | TGGCAGCAGCAGCATTATTC/ |  |  |  |  |
|  |  | CATTGTCGGCATGTCGTAGG |  |  |  |  |
| CO92-YPO1962 | *astC* | GCTACCTGCGGATAAAGAGTTC/ | CCCTCCGATGTTATTAC/ |  |  |  |
|  |  | TGGCACTGGTCAGCACATC | TTGCCCCCTTTAATCAGC |  |  |  |
| CO92-YPO1963 | *astA* |  | GCTTATTGCCCTGATTGCTG/ |  | GCTTATTGCCCTGATTGCTG/ |  |
|  |  |  | GTTCTATCCGAGCGGCGAG |  | GTTCTATCCGAGCGGCGAG |  |
| CO92-YPO2060 | *znuC* | GACATTCTGCCTGCACTCAC/ | AGCGGGCGTATTGATGTAAC/ | GGCGAGAGCCAAAAGTGACG |  | TCCCCCGGGATGGTGGTCATCAGTCGG/ |
|  |  | CCCTTGCGTTGGTTCATCC | GGCGAGAGCCAAAAGTGACG |  | GGCGAGAGCCAAAAGTGACG | CGCGGATCCAGTGTGGATTTTCCTGCG |
| CO92-YPO2061 | *znuA* | TCGGATGTCCAGCGGTTAC/ | CGCAGAGAAAGGGAAATATCG/ | ACCTCGGTTGGCAGCAC |  | TCCCCCGGGCTGTGGGCGGTTCAGTAAG/ |
|  |  | CCTCTTCAGCCTCATCATGTTC | AGGGATTTGCCAGTAAAAGTGC |  | AGGGATTTGCCAGTAAAAGTGC | CGCGGATCCGCCAGATTAAAGGGATTTG |
| CO92-YPO2374 | *rovA* | ATCGTTTACCACCAGAGCAATC/ | ATCCATCTGGCTATGTCG/ |  |  |  |
|  |  | AATCACGCCATCAACCTGTTC | TATACCGTCGATGCTAGG |  |  |  |
| CO92-YPO2665 | *ureA* | CAATGGAAGGAGCCAGAGATG/ |  |  |  |  |
|  |  | TCGTGTACCGTGACCAGAC |  |  |  |  |
| CO92-YPO2774 | *hisJ* | GCTAATCCCGTCGCTGAAAG/ |  |  |  |  |
|  |  | GTGTCGGCTCAATAGTGCTAC |  |  |  |  |
| CO92-YPO3041 | *narP* | GTCTTCAGCGACGAGGTG/ |  |  |  |  |
|  |  | TGTACTTTCACGGTTTCTTCTG |  |  |  |  |
| CO92-YPO3134 | *ykgM* | ACGATACCAGTGCGGATGC/ | TTATTGCTGAGCGTTATCC/ | TGTTCGCTCAGTGGCAATGG | TTATTGCTGAGCGTTATCC/ | CCGGAATTCAGAACGCCATCTTGATTG/ |
|  |  | TGGAAGCGTGCGGTACTG | TGTTCGCTCAGTGGCAATGG |  | TGTTCGCTCAGTGGCAATGG | CGCGGATCCTTCGCTCAGTGGCAATGG |
| CO92-YPO4018 | *cysM* | CGTGGCATAGTTGTGGTGAAG/ |  |  |  |  |
|  |  | CCATGTCGCTGTGAATAGATCG |  |  |  |  |
| *znuA* negative control | |  | CGGTTCAGTAAGGCTCGG/ |  |  |  |
|  |  |  | GTGGATCTCTGGTTCGTG |  |  |  |
| *gst* negative control | |  |  |  | GAGGAACCGAGAGAGAACG/ |  |
|  |  |  |  |  | GGGGTAATCTGCTCTCCTG |  |
| **primer** | | **sequence (5'-3')** | | | | |
| Zur-P-F | | GCGGGATCCATGAATATGATGAACC | | | | |
| Zur-P-R | | GCGAAGCTTTTACTTCTTTTTAACG | | | | |
| Zur-K-F | | ATGAATATGATGAACCCTATCAATCAGGAAAAGCTGCTCGGTTGTGTCTCAAAATCTCTG | | | | |
| Zur-K-R | | TTACTTCTTTTTAACGACGATGGAGTGGTCATGCTCACAAAAAGCCGCCGTCCCGTCAAG | | | | |
| Zur-I-F | | GTTGCTCACTGAGCGAACG | | | | |
| Kana-I-R | | TGGCTCATAACACCCCTTG | | | | |
| Kana-I-F | | CGTATTTCGTCTCGCTCAG | | | | |
| Zur-I-R | | ACTGGATCTGATCTGAGTG | | | | |
