# Supplementary material for: Characterization of Zur-dependent genes and direct Zur targets in Yersinia pestis
Source: BMC Microbiol. 2009 Jun 25;9:128. doi: 10.1186/1471-2180-9-128 (PMC2706843; doi:10.1186/1471-2180-9-128)
Supplement: Additional file 4 — A complete list of Zur-regulated genes. [file 1471-2180-9-128-S4.doc]

**A complete list of Zur-regulated genes**

| **Fold change** | **Gene ID** | **Gene name** | **Product** | **SAM report** | |
| --- | --- | --- | --- | --- | --- |
|  |  |  |  | **Score(d)** | **Denominator(s+s0)** |
| **aurine transport system** | |  |  |  |  |
| 2.3 | YPO0182 | *tauA* | putative taurine-binding periplasmic protein precursor | 2.32 | 0.52 |
| 2.16 | YPO0183 | *tauB* | putative taurine transport ATP-binding protein | 2.51 | 0.44 |
| 3.1 | YPO0184 | *tauC* | putative taurine transport system permease protein | 3.62 | 0.45 |
| 3.17 | YPO0185 | *tauD* | putative taurine dioxygenase | 2.62 | 0.64 |
| **Zinc uptake system** | |  |  |  |  |
| 2.99 | YPO2059 | *znuB* | high-affinity zinc uptake system membrane protein | 3.09 | 0.51 |
| 2.99 | YPO2060 | *znuC* | high-affinity zinc uptake system ATP-binding protein | 3.20 | 0.49 |
| 24.89 | YPO2061 | *znuA* | exported high-affinity zinc uptake system protein | 8.94 | 0.52 |
| 2.44 | YPO2062 |  | putative M23/M37 peptidase-family protein | 2.61 | 0.49 |
| **Oligopeptide transport system** | |  |  |  |  |
| -7.08 | YPO2182 | *oppA* | periplasmic oligopeptide-binding protein precursor | -5.51 | 0.51 |
| -4.53 | YPO2183 | *oppB* | oligopeptide transport system permease protein | -4.61 | 0.47 |
| -3.7 | YPO2184 | *oppC* | oligopeptide transport system permease protein | -4.31 | 0.44 |
| -4.48 | YPO2185 | *oppD* | oligopeptide transport ATP-binding protein | -4.71 | 0.46 |
| -4.04 | YPO2186 | *oppF* | oligopeptide transport ATP-binding protein | -4.36 | 0.46 |
| **Urease-related proteins** | |  |  |  |  |
| 5.42 | YPO2665 | *ureA* | urease gamma subunit | 5.23 | 0.47 |
| 3.63 | YPO2666 | *ureB* | urease beta subunit | 4.25 | 0.44 |
| 4.25 | YPO2667 | *ureC* | urease alpha subunit | 3.77 | 0.55 |
| 3.81 | YPO2668 | *ureE* | urease accessory protein | 4.44 | 0.43 |
| 3.76 | YPO2669 | *ureF* | urease accessory protein | 4.09 | 0.47 |
| 4.55 | YPO2670 | *ureG* | urease accessory protein | 4.83 | 0.45 |
| 3.34 | YPO2671 | *ureD* | urease accessory protein (pseudogene) | 3.79 | 0.46 |
| **Regulators** |  |  |  |  |  |
| -2.16 | YPO0315 |  | putative regulatory protein | -2.23 | 0.50 |
| -5.58 | YPO0712 | *fleR* | sigma-54 transcriptional regulatory protein | -5.24 | 0.47 |
| 74.18 | YPO2374 | *rovA* | MarR-family transcriptional regulatory protein | 12.54 | 0.50 |
| -2.68 | YPO2449 |  | putative LuxR-family regulatory protein | -2.86 | 0.50 |
| -4.42 | YPO3009 |  | putative two-component response regulator | -3.51 | 0.61 |
| 8.05 | YPO3041 | *narP* | nitrate/nitrite response regulator protein NarP | 4.06 | 0.74 |
| 2.52 | YPO3789 | *metR* | lysR-family transcriptional regulatory protein | 2.16 | 0.62 |
| **Exported or membrane proteins** | | | |  |  |
| 2.04 | YPO0130 |  | putative exported protein | 2.01 | 0.51 |
| 2.19 | YPO0337 |  | putative exported protein | 2.22 | 0.51 |
| -2.06 | YPO0702 |  | putative exported protein | -2.01 | 0.52 |
| -4.25 | YPO0987 |  | putative exported protein | -4.16 | 0.50 |
| -2.38 | YPO1388 |  | putative exported protein | -2.75 | 0.46 |
| -2.04 | YPO1442 |  | putative exported protein | -2.34 | 0.47 |
| -2.19 | YPO1718 |  | putative exported protein | -2.42 | 0.47 |
| -2.47 | YPO2155 |  | putative exported protein | -2.98 | 0.44 |
| -2.05 | YPO2262 |  | putative exported protein | -1.93 | 0.54 |
| 10.34 | YPO2674 |  | putative exported protein | 4.05 | 0.83 |
| -2.27 | YPO2732 | *lemA* | putative exported protein | -2.23 | 0.53 |
| 7.81 | YPO2902 |  | putative exported protein | 5.28 | 0.56 |
| -5.03 | YPO3152 | *ybaV* | putative exported protein | -4.16 | 0.56 |
| 2.88 | YPO3232 |  | putative exported protein | 3.03 | 0.50 |
| -3.51 | YPO1635 |  | putative lipoprotein | -3.11 | 0.65 |
| -2.24 | YPO1674 |  | putative lipoprotein | -2.47 | 0.47 |
| 8.99 | YPO2373 | *slyB* | putative lipoprotein | 6.84 | 0.46 |
| 3.35 | YPO2972 |  | putative lipoprotein | 3.76 | 0.46 |
| -9.18 | YPO3140 | *ybaY* | putative lipoprotein | -5.97 | 0.54 |
| 2.01 | YPO0590 | *hdeD* | putative membrane protein | 2.27 | 0.44 |
| -2.36 | YPO2801 |  | putative membrane protein | -2.64 | 0.47 |
| -6.93 | YPO3007 |  | putative membrane protein | -6.26 | 0.45 |
| 2.56 | YPO0079 | *sbp1* | exported sulfate-binding protein | 2.25 | 0.60 |
| -2.13 | YPO0251 |  | putative transmembrane transport protein | -2.33 | 0.47 |
| **Iron uptake** | |  |  |  |  |
| -2.02 | YPO1537 |  | putative iron-siderophore receptor | -1.93 | 0.53 |
| -2.7 | YPO1538 |  | putative siderophore biosynthetic enzyme | -2.90 | 0.50 |
| -3.84 | YPO3340 |  | putative exogenous ferric siderophore receptor (pseudogene) | -4.17 | 0.47 |
| -3.04 | YPO1906 | *fyuA* | pesticin/yersiniabactin receptor protein | -3.00 | 0.54 |
| -2.48 | YPO2958 | *sfuA* | iron(III)-binding periplasmic protein | -2.42 | 0.54 |
| **Chemotaxis and mobility** | |  |  |  |  |
| -2.7 | YPO0711 | *fliM* | putative flagellar motor switch protein | -2.81 | 0.58 |
| -2.56 | YPO0724 | *flgD* | putative basal-body rod modification protein | -2.35 | 0.58 |
| -4.77 | YPO0740 | *fliD* | putative flagellar hook-associated protein | -4.15 | 0.54 |
| **Degradation of small molecules and macromolecules** | | | |  |  |
| -2.6 | YPO0253 | *acs* | acetyl-coenzyme A synthetase | -2.33 | 0.59 |
| 34.02 | YPO1201 |  | putative amino acid decarboxylase | 10.61 | 0.48 |
| -2.91 | YPO1386 | *ansB* | putative L-asparaginase II precursor | -1.97 | 0.78 |
| -3 | YPO1962 | *ast* | succinylornithine aminotransferase | -3.51 | 0.45 |
| -3.68 | YPO1963 | *astA* | arginine N-succinyltransferase | -2.71 | 0.76 |
| -4.12 | YPO1964 | *astD* | succinylglutamic semialdehyde dehydrogenase | -4.17 | 0.49 |
| -3 | YPO1965 | *astB* | succinylarginine dihydrolase | -2.81 | 0.56 |
| -4.75 | YPO1231 | *pla2* | putative outer membrane-associated protease | -4.88 | 0.46 |
| -2.11 | YPO1683 |  | probable N-acetylmuramoyl-L-alanine amidase | -2.29 | 0.47 |
| -3.61 | YPO2803 | *bglB* | putative beta-glucosidase | -3.94 | 0.47 |
| **Transport/binding proteins** | |  |  |  |  |
| -5.41 | YPO2255 | *araF* | L-arabinose-binding periplasmic protein precursor | -3.44 | 0.71 |
| -2.79 | YPO2514 | *glnQ* | putative glutamine transport ATP-binding protein | -2.89 | 0.51 |
| -2.62 | YPO2774 | *hisJ* | histidine-binding periplasmic protein | -2.49 | 0.56 |
| 2.15 | YPO3624 | *ssuA* | putative aliphatic sulfonates binding protein | 2.50 | 0.44 |
| -2.33 | YPO2615 | *glnH* | putative amino acid-binding protein precursor | -2.67 | 0.46 |
| 2.9 | YPO2673 |  | putative nickel transport protein | 3.50 | 0.44 |
| 4.67 | YPO2675 |  | putative potassium channel protein | 4.99 | 0.45 |
| 3.7 | YPO1517 |  | putative sugar ABC transporter | 4.13 | 0.46 |
| -2.03 | YPO3697 | *treB* | PTS system, trehalose-specific IIBC component | -1.92 | 0.53 |
| 2.02 | YPO3908 |  | putative periplasmic protein precursor | 2.16 | 0.47 |
| -2.01 | YPO3992 | *dctA* | C4-dicarboxylate transport protein | -2.11 | 0.48 |
| 227.83 | YPO1343 |  | putative periplasmic binding protein | 13.02 | 0.60 |
| 45.76 | YPO1344 |  | FecCD transport family protein | 6.88 | 0.80 |
| 3.64 | YPO1346 |  | conserved hypothetical protein | 3.01 | 0.62 |
| 9.32 | YPO1347 |  | putative exported protein | 6.14 | 0.52 |
| 6.28 | YPO1348 |  | putative membrane protein | 4.50 | 0.57 |
| -2.36 | YPO1913 | *ybtP* | lipoprotein inner membrane ABC-transporter | -2.52 | 0.49 |
| 2.33 | YPO2660 |  | putative solute-binding protein | 2.34 | 0.52 |
| 2.54 | YPO2796 | *yapC* | putaive autotransporter protein | 2.34 | 0.58 |
| -2.24 | YPO2886 | *yapA* | putative autotransporter protein | -1.88 | 0.62 |
| -2.22 | YPO3650 |  | putative metabolite transport protein | -1.91 | 0.60 |
| **Pathogenicity** | |  |  |  |  |
| 2.18 | YPO0339 |  | enhancing factor (viral) | 2.31 | 0.49 |
| 23.91 | YPO1301 | *psaE* | putative regulatory protein | 8.29 | 0.55 |
| 69.55 | YPO1302 | *psaF* | putative membrane protein | 10.19 | 0.60 |
| 6.18 | YPO1303 | *psaA* | pH 6 antigen precursor (antigen 4) (adhesin) | 4.32 | 0.61 |
| 2.09 | YPO1793 | *inv* | invasin (pseudogene) | 2.31 | 0.46 |
| 20.03 | YPO2190 | *ail* | attachment invasion locus protein precursor | 5.30 | 0.78 |
| **Biosynthesis and metabolism** | |  |  | |  |
| 4.35 | YPO0117 | *metF* | 5,10-methylenetetrahydrofolate reductase | 4.48 | 0.47 |
| 2.92 | YPO3727 | *metA* | homoserine O-succinyltransferase | 2.94 | 0.53 |
| 8.24 | YPO3788 | *metE* | 5-methyltetrahydropteroyltriglutamate--homocysteine methyltransferase | 6.85 | 0.44 |
| -3.05 | YPO2409 | *ppsA* | phosphoenolpyruvate synthase | -3.08 | 0.52 |
| -2.72 | YPO3036 | *napC* | CysB | -3.03 | 0.48 |
| -3.26 | YPO3342 | *yhjA* | putative cytochrome C peroxidase | -2.80 | 0.61 |
| -2.05 | YPO0793 | *aas* | Aas bifunctional protein | -2.25 | 0.46 |
| 2.29 | YPO2390 | *cfa* | cyclopropane-fatty-acyl-phospholipid synthase | 2.40 | 0.50 |
| -3.59 | YPO3198 | *ggt* | gamma-glutamyltranspeptidase precursor (pseudogene) | -3.56 | 0.52 |
| 2.14 | YPO1105 |  | DNA repair protein RecN | 2.10 | 0.53 |
| 13.14 | YPO3134 | *ykgM* | putative ribosomal protein | 7.40 | 0.50 |
| 59.43 | YPO3135 | *rpmJ2* | putative ribosomal protein L36 | 11.63 | 0.51 |
| **Stress responsive genes** | |  |  |  |  |
| -2.51 | YPO2234 | *cstA* | putative carbon starvation protein A | -2.20 | 0.60 |
| 2.33 | YPO2510 | *dps* | putative DNA-binding protein | 2.68 | 0.46 |
| **Others** |  |  |  |  |  |
| -2.12 | YPO1096 |  | putative phage protein | -2.20 | 0.49 |
| -2.48 | YPO2103 |  | putative phage terminase (pseudogene) | -2.06 | 0.64 |
| -2.39 | YPO2943 |  | outer membrane usher protein (pseudogene) | -2.47 | 0.51 |
| **Unknown** |  |  |  |  |  |
| -4.59 | YPO0499 |  | hypothetical protein | -4.84 | 0.45 |
| -4.37 | YPO0500 |  | conserved hypothetical protein | -4.34 | 0.49 |
| -4.49 | YPO0501 |  | conserved hypothetical protein | -4.86 | 0.45 |
| -5.88 | YPO0502 |  | conserved hypothetical protein | -5.68 | 0.45 |
| -5.23 | YPO0503 |  | conserved hypothetical protein | -4.87 | 0.49 |
| -7.44 | YPO0504 |  | conserved hypothetical protein | -4.67 | 0.62 |
| -2.24 | YPO0506 | *clpB* | putative Clp ATPase | -2.29 | 0.51 |
| -2.84 | YPO0507 |  | conserved hypothetical protein | -2.36 | 0.64 |
| -5.13 | YPO0508 |  | hypothetical protein | -4.09 | 0.58 |
| -5.16 | YPO0509 |  | hypothetical protein | -4.05 | 0.58 |
| -2.51 | YPO0510 |  | hypothetical protein | -2.31 | 0.58 |
| -3.09 | YPO0511 |  | hypothetical protein | -2.57 | 0.63 |
| -5.7 | YPO0511a |  | hypothetical protein | -3.99 | 0.63 |
| -4.41 | YPO0512 |  | putative lipoprotein | -4.16 | 0.51 |
| -4.78 | YPO0513 |  | conserved hypothetical protein | -3.45 | 0.65 |
| -3.39 | YPO0514 |  | putative OmpA-family membrane protein | -3.17 | 0.56 |
| -2.67 | YPO0515 |  | putative membrane protein | -2.64 | 0.54 |
| -3.93 | YPO0516 |  | hypothetical protein | -4.21 | 0.47 |
| -2.09 | YPO0623 |  | putative aminotransferase | -1.95 | 0.54 |
| 8.12 | YPO0625 |  | hypothetical protein | 5.61 | 0.54 |
| 3.76 | YPO0626 |  | conserved hypothetical protein | 3.98 | 0.48 |
| 4.69 | YPO0627 |  | putative translational inhibitor protein | 5.03 | 0.44 |
| 4.56 | YPO0628 |  | putative translational inhibitor protein | 3.84 | 0.57 |
| -4.18 | YPO1989 |  | putative exported protein | -4.36 | 0.47 |
| -5.07 | YPO1992 |  | conserved hypothetical protein | -3.16 | 0.74 |
| -2.43 | YPO1994 |  | hypothetical protein | -2.69 | 0.48 |
| -2.27 | YPO1995 |  | hypothetical protein | -2.73 | 0.43 |
| -2.47 | YPO1996 |  | hypothetical protein | -2.95 | 0.44 |
| 87.97 | YPO4018 | *cysM* | pyridoxal-phosphate dependent protein | 11.58 | 0.56 |
| 43.88 | YPO4019 |  | putative phosphoribosyl transferase protein | 7.98 | 0.68 |
| 16.93 | YPO4020 |  | putative membrane protein | 6.49 | 0.63 |
| 9.73 | YPO4021 |  | hypothetical protein | 6.55 | 0.50 |
| -2.41 | YPO0100 |  | hypothetical protein | -2.38 | 0.54 |
| 2.26 | YPO0431 | *osmY* | osmotically inducible protein Y | 2.66 | 0.44 |
| 2.01 | YPO0601 |  | hypothetical protein | 1.99 | 0.51 |
| -2.51 | YPO0639 |  | hypothetical protein | -2.62 | 0.51 |
| -2.51 | YPO0749 |  | hypothetical protein | -2.61 | 0.51 |
| -2.92 | YPO1192 |  | conserved hypothetical protein | -2.64 | 0.56 |
| -2.48 | YPO1255 |  | hypothetical protein | -2.64 | 0.50 |
| -2.38 | YPO1437 |  | conserved hypothetical protein | -2.29 | 0.55 |
| 2.25 | YPO1516 |  | hypothetical protein | 2.00 | 0.58 |
| -2.42 | YPO2467 |  | conserved hypothetical protein | -2.65 | 0.48 |
| 3.69 | YPO3982 |  | hypothetical protein | 2.80 | 0.67 |

mRNA level of each gene was compared between WT and *Δzur* upon exposure to zinc rich conditions. The data are presented as the mean change of mRNA level for each gene under the paired growth conditions. The positive number stands for fold increased, while minus decreased.
