# Supplementary figures and images for: Characterization of Zur-dependent genes and direct Zur targets in Yersinia pestis
Source: BMC Microbiol. 2009 Jun 25;9:128. doi: 10.1186/1471-2180-9-128 (PMC2706843; doi:10.1186/1471-2180-9-128)

**
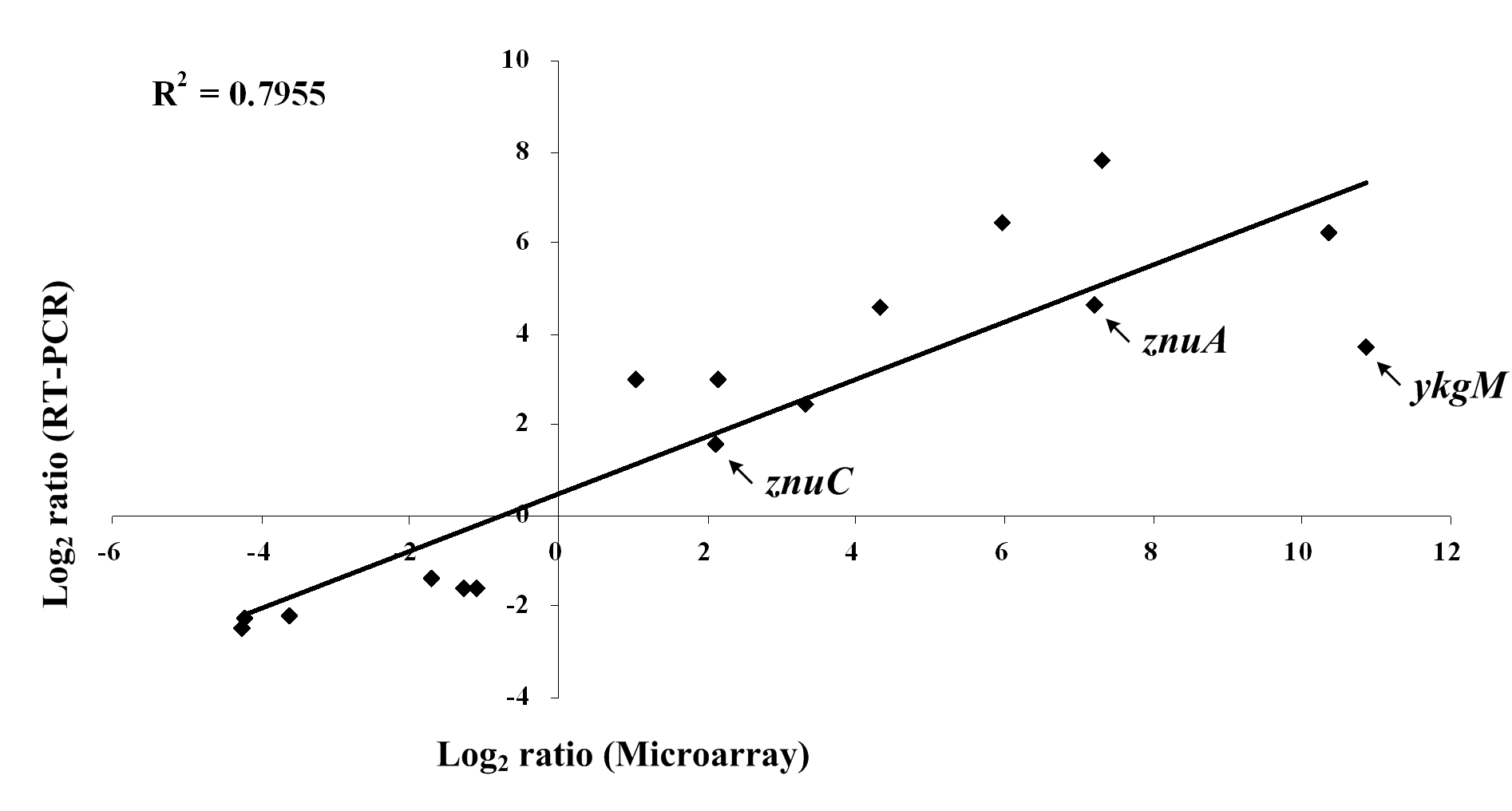
**

Supplement: Additional file 5 — Comparison of transcription measurements by microarray and real-time PCR assays. The relative transcriptional levels for 17 genes selected from Supplementary Table S1 were determined by real-time RT-PCR. The log2 values were plotted against the microarray data log2 values. The correlation coefficient (R2) for comparison of the two datasets is 0.796. [file 1471-2180-9-128-S5.doc]
